# Medical Appointments

**Published:** 1850-07

**Authors:** 


### MEDICAL APPOINTMENTS.

Dr. Joseph Carson has been appointed Professor of Materia Medica and Pharmacy in the University of Pennsylvania, in place of Dr. Wood, transferred to the Chair of Practice.

The name of Dr. Carson has long been associated with Materia Medica and Pharmacy both as a teacher and writer. Many of the most valuable contributions to the Journal of Pharmacy, and the American reprint of Pereira's Materia Medica, were from his pen, and the splendid work on Medical Botany, of which he is the author, has stamped him as a proficient in his department. We congratulate the friends of the University on the appointment.

Dr. Daniel Drake has resigned the Chair of Theory and Practice of Medicine in the Medical College of Ohio.

Dr. John Bell has received and accepted the appointment of Professor of the Theory and Practice of Medicine in the Medical College of Ohio in place of Dr. Drake, resigned.

Although his numerous friends in Philadelphia will deeply regret his departure from among them, to the profession in Cincinnati it is a source of congratulation that they secured the services of so able and learned, and, withal, so high-toned a gentleman.

Dr. S. Hanbury Smith, the able editor of the Ohio Medical and Surgical Journal, has been appointed Superintendent of the Ohio State Lunatic Asylum, at Columbus, in place of Dr. Awl resigned.

